# Associations of Objectively Assessed Physical Activity and Sedentary Time with All-Cause Mortality in US Adults: The NHANES Study

**DOI:** 10.1371/journal.pone.0119591

**Published:** 2015-03-13

**Authors:** Daniela Schmid, Cristian Ricci, Michael F. Leitzmann

**Affiliations:** Department of Epidemiology and Preventive Medicine, University of Regensburg, Franz-Josef-Strauss-Allee 11, 93053, Regensburg, Germany; Pennington Biomedical Research Center, UNITED STATES

## Abstract

**Background:**

Sedentary behavior is related to increased mortality risk. Whether such elevated risk can be offset by enhanced physical activity has not been examined using accelerometry data.

**Materials and Methods:**

We examined the relations of sedentary time and physical activity to mortality from any cause using accelerometry data among 1,677 women and men aged 50 years or older from the National Health and Nutrition Examination Survey (NHANES) 2003–2004 cycle with follow-up through December 31, 2006.

**Results:**

During an average follow-up of 34.67 months and 4,845.42 person-years, 112 deaths occurred. In multivariate Cox proportional hazard models, greater sedentary time (≥ median of 8.60 hours/day) was associated with increased risk of mortality from any cause (relative risk (RR) = 2.03; 95% confidence interval (CI) = 1.09-3.81). Low level of moderate to vigorous physical activity (< median of 6.60 minutes/day) was also related to enhanced all-cause mortality risk (RR = 3.30; 95% CI = 1.33-8.17). In combined analyses, greater time spent sedentary and low levels of moderate to vigorous physical activity predicted a substantially elevated all-cause mortality risk. As compared with the combination of a low sedentary level and a high level of moderate to vigorous physical activity, the risks of mortality from all causes were 4.38 (95% CI = 1.26-15.16) for low levels of both sedentary time and physical activity, 2.79 (95% CI = 0.77-10.12) for greater time spent sedentary and high physical activity level, and 7.79 (95% CI = 2.26-26.82) for greater time spent sedentary and low physical activity level. The interaction term between sedentary time and moderate to vigorous physical activity was not statistically significant (p = 0.508).

**Conclusions:**

Both high levels of sedentary time and low levels of moderate to vigorous physical activity are strong and independent predictors of early death from any cause. Whether a high physical activity level removes the increased risk of all-cause mortality related to sedentariness requires further investigation.

## Introduction

High amounts of time spent sedentary and low levels of physical activity are major public health concerns in industrialized countries. On average, United States (U.S.) adults spend 50 to 60% of their day in sedentary behaviors [1] and the vast majority does not participate in regular physical activity [2]. A growing body of evidence suggests that high levels of sedentary time represent a risk factor for premature mortality [3–14]. A recent meta-analysis reported a 2% increased risk of mortality for each one hour per day increase in self-reported total sitting time and a 34% higher mortality risk for those who indicated sitting 10 hours per day [15]. By comparison, physical activity is known to prevent premature mortality, with a recent meta-analysis estimating that increased self-reported physical activity is related to a 35% reduced risk of mortality [16].

It is of particular importance to gain insight into the risk of mortality associated with excessive sedentary time in individuals who are otherwise physically active to determine the optimal balance between time spent in sedentary behaviors and physical activity for longevity. Should individuals who sit for most of the day carry health risk despite high physical activity levels, then future physical activity guidelines may need to be expanded to include recommendations on daily sedentary time. However, only three studies have examined the joint relations of sedentary time and physical activity to risk of mortality to date [4, 6, 8]. Two of those studies observed that high physical activity levels did not fully negate the apparent deleterious effect of prolonged total sedentary time or television (TV) viewing time on mortality [6, 8]. The third study reported a positive dose-response relation between sedentary time and mortality that was similar in both physically active and physically inactive individuals [4]. These investigations are limited by their use of self-report methods, which bear the potential for reporting errors of sedentary behavior and physical activity [17–21].

To our knowledge, no study has examined the joint relations of objectively measured sedentary time and physical activity to risk of mortality. To address this critical issue, we used accelerometry data from a nationally representative sample of the U.S. population to examine whether higher levels of physical activity can alleviate the apparent adverse effect of time spent sedentary on early mortality from any cause.

## Methods

### Ethics Statement

The National Center for Health Statistics Research Ethics Review Board approved the survey protocols, and written informed consent was obtained for all individuals.

### Study population

The present study was based on data from the 2003–2004 NHANES cycle that used a multistage probability sampling design to generate a sample of participants representative of the civilian non-institutionalized U.S. population [22]. For the purposes of our analyses, we included accelerometry data from individuals aged 50 years or older who achieved a minimum of twelve hours of monitor wear on one or more days. From 2,510 eligible participants, we excluded 833 persons for the following reasons: missing or invalid accelerometry data (n = 643), missing information on survey weight (n = 183), missing data regarding mortality status or duration of follow-up (n = 6), or unknown information about mortality cause (n = 1), leaving 1,677 participants for analysis. Information on demographics, socioeconomic status, and medical history was collected during an in-home interview [22]. Study participants were further invited to a mobile examination center (MEC) for standardized clinical examinations including anthropometric measurements. Data from participants of the NHANES 2003–2004 survey were linked to death certificate data from the National Death Index. Additional sources of follow-up for mortality included the Social Security Administration, the Centers for Medicare and Medicaid Services, and death certificates [23]. Person-years of follow-up were calculated from the date of examination until date of death or censoring on December 31, 2006, whichever occurred first.

### Physical activity and sedentary behavior measures

Physical activity was assessed using the uniaxial ActiGraph AM-7164 accelerometer (ActiGraph, Ft. Walton Beach, FL). The physical activity monitor was positioned on the waist by a belt for up to seven consecutive days. Wear time was determined by subtracting non-wear time from 24 hours [24]. Non-wear time was defined as an interval of at least 60 consecutive minutes of zero counts, allowing for intervals of 1–2 minutes of relatively low counts (i.e., 1–100 counts) [24–27]. To define sedentary time and moderate to vigorous physical activity, we used cut-points from published calibration studies [25, 26, 28–30]. Specifically, sedentary time was defined as less than 100 counts per minute [30]. Moderate to vigorous physical activity was defined as a minimum of 2,020 counts per minute, which represents the weighted average cut-point for moderate to vigorous physical activity from published calibration studies [25, 26, 28, 29] as described by Metzger et al. [31].

### Statistical analysis

We used Cox proportional hazards models to estimate relative risks (RRs) and corresponding 95% confidence intervals (CIs) of mortality. Schoenfeld´s residuals [32] were used to verify the proportional hazards assumption. Sample weights were included in the models as weight variables to reduce the variance in the estimation procedure, to avoid the effect of differential probabilities of selection among subgroups, and to compensate for exclusion of sampling areas in the sampling frame. To estimate the independent effects of sedentary time and moderate to vigorous physical activity on mortality from any cause, we mutually adjusted for sedentary time and moderate to vigorous physical activity. We examined risk of those exposures relative to low-risk referent categories (i.e., low sedentary time, high moderate to vigorous physical activity). We dichotomized sedentary time and moderate to vigorous physical activity at their medians to yield sufficient numbers of participants for analysis and to facilitate the complex nature of joint associations. The multiplicative interaction between sedentary time and moderate to vigorous physical activity was evaluated using a Wald test.

The basic model was adjusted for age and sex. An additional model was further adjusted for education (less than high school, high school, more than high school), ethnicity (Caucasian, non-Hispanic black, Hispanic), smoking habits (current, former, never), light physical activity (<310.5 min/day, ≥310.5 min/day; defined as 100 to 2019 accelerometer counts per minute), alcohol consumption (current, former, never), history of chronic diseases (diabetes, coronary heart disease, congestive heart failure, stroke, cancer), and mobility limitations (difficulty walking a quarter of a mile or walking up ten steps without resting). A third model was mutually adjusted for sedentary time (continuous) and moderate to vigorous physical activity (continuous). In a fourth model, we additionally adjusted for body mass index (BMI) modeled as a categorical variable according to the World Health Organization (WHO) cut-points (<18.5, 18.5–24.9, 25.0–29.9, ≥30.0 kg/m²). Separate categories were created for covariates with missing values. In sensitivity analyses, potential reverse causality was assessed by excluding persons with a history of chronic diseases, those with mobility limitations, or deaths that occurred during the first year of follow up. We also examined whether findings were altered when we selected individuals with four or more valid days of accelerometry data instead of one or more valid day. To explore whether the cut-points selected for the accelerometer counts affected our results, in a further sensitivity analysis we varied the cut-points for sedentary time (<50 and <150 counts per minute) and moderate to vigorous physical activity (>1,500 and >2,500 counts per minute).

The type I error rate was set at 0.05 and all tests and confidence limits were two-tailed. All statistical analyses were performed using SAS release 9.2 and the Cox proportional models were conducted using the SURVEYPHREG procedure (SAS Institute Inc., Cary, NC).

## Results

During a median follow-up time of 34.67 months, the analytic cohort of 1,677 participants accumulated 4,845.42 person-years of follow-up, among which 112 deaths occurred. Age-standardized baseline characteristics of the cohort according to the medians of sedentary time (8.60 h/d) and moderate to vigorous physical activity (6.60 min/d) are shown in Table 1. The median age of the participants was approximately 67 years. Overall, subjects with sedentary time above the median were more likely to be Caucasian and to have a history of diabetes, cardiovascular disease (CVD), cancer, and mobility limitations than those with time spent sedentary below the median. Participants with a higher moderate to vigorous physical activity level were less likely to have formerly smoked and to have a history of diabetes, CVD, cancer, and mobility limitations than those with a lower moderate to vigorous physical activity level.

**Table 1:** Age-standardized baseline characteristics of the study population by medians of sedentary time (8.60 hours per day) and moderate to vigorous physical activity (6.60 minutes per day).

	Sedentary time (h/d)		Moderate to vigorous physical activity (min/d)	
	<8.60	≥8.60	<6.60	≥6.60
Number of individuals	838	839	836	841
Mean age at baseline (years)	64.9	69.5	71.7	62.7
Sex (%)				
Men	49.6	48.9	47.7	48.2
Women	50.4	51.1	52.3	51.8
Education (%)				
<High school	36.4	46.7	36.8	43.4
High school	22.2	25.4	26.1	22.8
>High school	41.0	27.6	36.7	33.4
Ethnicity (%)				
Caucasian	56.9	63.8	62.1	58.0
Black	14.2	14.8	16.9	13.6
Hispanic	26.4	16.5	17.4	24.7
Missing	2.49	4.94	3.67	3.78
BMI (%)				
<18.5 kg/m^2^	0.7	0.7	0.7	0.7
18.5–24.9 kg/m^2^	30.2	31.2	29.6	31.8
25.0–29.9 kg/m^2^	40.6	38.2	37.1	41.7
≥30.0 kg/m^2^	26.2	28.1	30.6	23.8
Missing	2.3	1.8	2.0	2.0
Light physical activity (%)				
<310.5 min/d	27.8	72.1	69.4	30.7
≥310.5 min/d	72.2	27.9	30.6	69.3
Alcohol consumption (%)				
Current	52.6	49.7	44.6	55.0
Former	28.8	30.0	34.1	27.2
Never	14.9	16.3	18.0	13.9
Missing	3.71	4.02	3.38	3.93
Smoking status (%)				
Current	40.3	38.8	37.0	39.9
Former	13.9	14.9	20.1	11.4
Never	45.8	46.4	42.9	48.7
Chronic disease history				
Diabetes (%)	13.7	19.2	23.1	11.5
Coronary heart disease (%)	8.7	9.4	9.9	7.3
Congestive heart failure (%)	4.7	6.8	8.0	3.1
Stroke (%)	5.7	6.9	8.1	4.0
Cancer (%)	14.4	18.2	18.0	16.8
Difficulty walking a quarter of a mile (%)	14.7	18.1	21.7	10.0
Difficulty walking up ten steps (%)	10.5	14.1	17.2	7.6

In age- and sex-adjusted analyses, a high versus low level of sedentary time was associated with a statistically significant increased risk of mortality from any cause (RR = 3.25; 95% CI = 1.88–5.62) (Table 2). Adjustment for potential confounding variables attenuated the relation but it remained statistically significant (RR = 2.08; 95% CI = 1.13–3.82). Additional control for moderate to vigorous physical activity did not further materially change the result (RR = 2.03; 95% CI = 1.09–3.18). Further adjustment for BMI also had little impact on the risk estimate (RR = 1.96; 95% CI = 1.10–3.52).

**Table 2:** Age- and sex-, and multivariate-adjusted relative risks (RRs) and 95% confidence intervals (CIs)* of mortality from any cause according to sedentary time and moderate to vigorous physical activity.

	Total mortality	
*Sedentary time*		
	Sedentary time <8.60 h/d	Sedentary time ≥8.60 h/d
Deaths, n	26	86
Person-yrs	2466.3	2379.1
Age- and sex-adjusted RR (95% CI)	1.0 (ref.)	3.25 (1.88–5.62)
Multivariate-adjusted RR (95% CI)	1.0 (ref.)	2.08 (1.13–3.82)
Multivariate and MVPA-adjusted RR (95% CI)	1.0 (ref.)	2.03 (1.09–3.81)
Multivariate and MVPA- and BMI-adjusted RR (95% CI)	1.0 (ref.)	1.96 (1.10–3.52)
*Moderate to vigorous physical activity*		
	Moderate to vigorous physical activity ≥ 6.60 min/d	Moderate to vigorous physical activity<6.60 min/d
Deaths, n	14	98
Person-yrs	2484.6	2360.8
Age- and sex-adjusted RR (95% CI)	1.0 (ref.)	6.32 (2.69–14.88)
Multivariate-adjusted RR (95% CI)	1.0 (ref.)	3.44 (1.39–8.50)
Multivariate and sedentary time-adjusted RR (95% CI)	1.0 (ref.)	3.30 (1.33–8.17)
Multivariate and sedentary time- and BMI-adjusted RR (95% CI)	1.0 (ref.)	3.28 (1.33–8.13)
*Combined sedentary time and moderate to vigorous physical activity*		
	Moderate to vigorous physical activity ≥ 6.60 min/d	Moderate to vigorous physical activity<6.60 min/d
Sedentary time <8.60 h/d		
Deaths, n	6	20
Person-yrs	1619.3	847.1
Age- and sex-adjusted RR (95% CI)	1.0 (ref.)	5.75 (1.79–18.48)
Multivariate-adjusted RR (95% CI)	1.0 (ref.)	4.38 (1.26–15.16)
Multivariate and BMI-adjusted RR (95% CI)	1.0 (ref.)	4.30 (1.23–15.05)
Sedentary time ≥8.60 h/d		
Deaths, n	8	78
Person-yrs	865.3	1513.8
Age- and sex-adjusted RR (95% CI)	2.87 (0.83–9.88)	15.39 (4.86–48.70)
Multivariate-adjusted RR (95% CI)	2.79 (0.77–10.12)	7.79 (2.26–26.82)
Multivariate and BMI-adjusted RR (95% CI)	2.79 (0.77–10.21)	7.82 (2.24–27.24)

*Multivariate analyses are adjusted for age, sex, education, ethnicity, smoking, light physical activity, alcohol consumption, history of diabetes, history of cardiovascular disease (coronary heart disease, congestive heart failure, stroke), history of cancer, and mobility limitations.

MVPA = moderate to vigorous physical activity; BMI = body mass index; sedentary time and moderate to vigorous physical activity were mutually adjusted.

A low versus high level of moderate to vigorous physical activity was associated with a statistically significant increased risk of mortality from any cause in the age- and sex-adjusted model and confidence limits were wide (RR = 6.32; 95% CI = 2.69–14.88). Multivariate adjustment strongly attenuated the relation but it persisted to be statistically significant (RR = 3.44; 95% CI = 1.39–8.50) (Table 2). Additional inclusion of sedentary time in the model had little impact (RR = 3.30; 95% CI = 1.33–8.17) as did subsequent adjustment for BMI (RR = 3.28; 95% CI = 1.33–8.13).

We next examined the combined relations of sedentary time and moderate to vigorous physical activity to all-cause mortality (Table 2). As compared with the combination of low level of sedentary time and high level of moderate to vigorous physical activity, the multivariate relative risk of all-cause mortality for the combination of greater time spent sedentary and low level of moderate to vigorous physical activity was 7.79 (95% CI = 2.26–26.82). That association was not substantially altered after additional adjustment for BMI (RR = 7.82; 95% CI = 2.24–27.24). The relative risk of all-cause mortality was 4.38 (95% CI = 1.26–15.16) for the combination of low levels of both sedentary time and moderate to vigorous physical activity and it was 2.79 (95% CI = 0.77–10.12) for the combination of greater sedentary time and high level of moderate to vigorous physical activity. The interaction term between sedentary time and moderate to vigorous physical activity was not statistically significant (p = 0.508).

In sensitivity analyses, exclusion of persons with a history of diabetes or cancer resulted in a loss of statistical significance of the relations of time spent sedentary and moderate to vigorous physical activity to all-cause mortality. By comparison, exclusion of subjects with a history of CVD or those with mobility limitations did not affect the risk estimates for sedentary time or moderate to vigorous physical activity (Table 3). When we excluded deaths that occurred during the first year of follow-up, the independent association of sedentary time with all-cause mortality was attenuated (RR = 1.59; 95% CI = 0.84–3.03) but the relation of low level of moderate to vigorous physical activity to all-cause mortality remained evident (RR = 3.06; 95% CI = 1.13–8.26).

**Table 3:** Multivariate relative risks (RRs) and 95% confidence intervals (CIs)* of mortality from any cause according to sedentary time or moderate to vigorous physical activity among all participants and after exclusion of participants with a history of chronic diseases or mobility limitations, or deaths occurring within the first year of follow-up.

	Total mortality					
	Deaths, n	Person-yrs	RR (95% CI)	Deaths, n	Person-yrs	RR (95% CI)
**Sedentary time (h/d)**		**<8.60**			**≥8.60**	
Multivariate-adjusted^a^	25	2115.3	1.0 (ref.)	62	1934.8	1.38 (0.72–2.65)
Multivariate-adjusted^b^	15	2141.5	1.0 (ref.)	53	1917.2	2.53 (1.05–6.06)
Multivariate-adjusted^c^	20	2134.8	1.0 (ref.)	55	1923.7	1.66 (0.81–3.42)
Multivariate-adjusted^d^	19	2052.3	1.0 (ref.)	63	1820.9	2.17 (1.13–4.14)
Multivariate-adjusted^e^	21	2463.0	1.0 (ref.)	59	2362.8	1.59 (0.84–3.03)
**Moderate to vigorous physical activity (min/d)**		**≥6.60**			**<6.60**	
Multivariate-adjusted^a^	13	1854.8	1.0 (ref.)	74	2195.3	2.46 (0.87–7.00)
Multivariate-adjusted^b^	9	1839.8	1.0 (ref.)	59	2218.8	3.72 (1.28–10.77)
Multivariate-adjusted^c^	12	1888.8	1.0 (ref.)	63	2169.7	2.22 (0.73–6.74)
Multivariate-adjusted^d^	10	1695.8	1.0 (ref.)	72	2177.4	3.63 (1.48–8.88)
Multivariate-adjusted^e^	11	2344.0	1.0 (ref.)	69	2481.8	3.06 (1.13–8.26)

*Adjusted for age, sex, education, ethnicity, smoking, light physical activity, alcohol consumption, history of diabetes, history of cardiovascular disease (coronary heart disease, congestive heart failure, stroke), history of cancer, and mobility limitations. Sedentary time and moderate to vigorous physical activity were mutually adjusted.

a) Participants with history of diabetes excluded.

b) Participants with history of cardiovascular disease events excluded.

c) Participants with history of cancer excluded.

d) Participants with mobility limitations excluded.

e) Participants who died within the first year of follow-up excluded.

In further sensitivity analyses, the combination of greater time spent sedentary and low level of moderate to vigorous physical activity remained positively associated with risk of early mortality from any cause even after excluding participants with a history of diabetes (RR = 4.55; 95% CI = 1.15–18.02), CVD (RR = 13.50; 95% CI = 2.80–65.13), cancer (RR = 4.32; 95% CI = 1.04–17.89), those with mobility limitations (RR = 8.37; 95% CI = 1.98–35.27), or excluding deaths that occurred during the first year of follow up (RR = 5.40; 95% CI = 1.46–19.98) (Table 4).

**Table 4:** Multivariate relative risks (RRs) and 95% confidence intervals (CI)* of mortality from any cause according to joint categories of sedentary time and moderate to vigorous physical activity among all participants and after exclusion of participants with a history of chronic diseases or mobility limitations, or deaths occurring within the first year of follow-up.

	Total mortality					
	Moderate to vigorous physical activity					
	≥6.60 min/d			<6.60 min/d		
Sedentary time <8.60 h/d	Person-yrs	Deaths, n	RR (95% CI)	Person-yrs	Deaths, n	RR (95% CI)
Multivariate-adjusted^a^	1427.0	6	1.0 (ref.)	688.3	19	3.87 (1.01–14.83)
Multivariate-adjusted^b^	1457.2	4	1.0 (ref.)	684.3	11	5.98 (1.25–28.67)
Multivariate-adjusted^c^	1448.3	6	1.0 (ref.)	686.5	14	2.84 (0.71–11.31)
Multivariate-adjusted^d^	1412.7	4	1.0 (ref.)	639.7	15	3.91 (0.91–16.81)
Multivariate-adjusted^e^	1618.3	5	1.0 (ref.)	844.8	16	3.53 (0.94–13.19)
**Sedentary time ≥8.60 h/d**						
Multivariate-adjusted^a^	768.3	7	2.45 (0.67–9.00)	1166.5	55	4.55 (1.15–18.02)
Multivariate-adjusted^b^	761.7	5	4.38 (0.87–22.08)	1155.5	48	13.50 (2.80–65.13)
Multivariate-adjusted^c^	721.3	6	2.21 (0.54–9.02)	1202.3	49	4.32 (1.04–17.89)
Multivariate-adjusted^d^	764.8	6	2.37 (0.46–12.22)	1056.2	57	8.37 (1.98–35.27)
Multivariate-adjusted^e^	863.6	6	1.88 (0.47–7.52)	1499.3	53	5.40 (1.46–19.98)

*Adjusted for age, sex, education, ethnicity, smoking, light physical activity, alcohol consumption, history of diabetes, history of cardiovascular disease (coronary heart disease, congestive heart failure, stroke), history of cancer, and mobility limitations.

a) Participants with history of diabetes excluded.

b) Participants with history of cardiovascular disease events excluded.

c) Participants with history of cancer excluded.

d) Participants with mobility limitations excluded.

e) Participants who died within the first year of follow-up excluded.

Our findings were not materially altered when we selected individuals with four or more valid days of accelerometry data instead of one or more valid day, with a relative risk of all-cause mortality for the combination of greater time spent sedentary and low level of moderate to vigorous physical activity of 8.10 (95% CI = 2.31–28.11). Also, our results were not sensitive to the selection of different cut-points for the accelerometer counts, with all-cause mortality risk for the combination of greater time spent sedentary and low level of moderate to vigorous physical activity ranging from 5.29 (95% CI = 1.32–21.33) to 8.15 (95% CI = 2.27–29.13).

## Discussion

In our study of objectively assessed sedentary time and physical activity, we found that both high levels of sedentary time and low levels of vigorous physical activity were independent predictors of early mortality from any cause. Greater time spent sedentary remained associated with increased all-cause mortality risk even among those categorized as having a high level of moderate to vigorous physical activity, but that relation was not statistically significant. Thus, we were unable to conclusively determine whether a high level of physical activity counteracts the increased risk of mortality conferred by greater time spent sedentary.

In our analyses, the strength and direction of the combined association of greater time spent sedentary and low level of moderate to vigorous physical activity with early mortality remained evident after accounting for pre-existing chronic diseases or mobility limitations, or disregarding deaths that occurred during the first year of follow-up. However, because follow-up time was limited in our study, we cannot rule out that the observed association was partly due to reverse causation, which could have occurred if pre-existing illness caused participants to increase their sedentary time or decrease their physical activity level. Mutual adjustment for sedentary time and physical activity did not markedly affect the associations with early mortality, suggesting that the relations of these exposures to mortality are independent from each other.

While residual confounding by obesity may in theory contribute to the positive association between sedentary time and mortality, in our study the association with sedentary time was not essentially altered after controlling for BMI. This finding is in accordance with those from other studies [3, 4, 7, 8, 12, 13], stressing a role of sedentary behavior for mortality risk that is independent from adiposity.

Three previous studies based on self-administered questionnaires investigated the joint association of sedentary behavior and physical activity with total mortality and found that high physical activity levels did not counteract the apparent deleterious effects of greater time spent sedentary on mortality risk [4, 6, 8]. One study revealed 94% and 48% increased death rates among women and men, respectively, comparing high levels of sedentary time and low levels of moderate to vigorous physical activity with low levels of sedentary time and high levels of moderate to vigorous physical activity [6]. Another study found a 40% increased risk of mortality in individuals who reported sitting almost all of the time versus almost none of the time even among those who were physically active during leisure time [4]. Similarly, a third study reported that even among participants engaging in moderate to vigorous physical activity seven hours per week, TV viewing for seven hours per day remained associated with a 1.5-fold increased risk of mortality compared to TV viewing less than one hour per day [8]. Self-reported questionnaires bear the potential for measurement error [17–21] and may have resulted in attenuation of the strength of the risk estimates and underestimation of the true effects of sedentary behavior and physical activity on mortality risk in previous studies. Measurement error could also have produced an overestimation of the associations.

The findings of the present study confirm the results of a previous study examining the relation between objectively-assessed sedentary time and risk of mortality from any cause in the NHANES population [9]. The novelty of our study is that we investigated the joint relations of sedentary time and physical activity to mortality.

The exact mechanisms linking high sedentariness to enhanced risk of mortality remain to be elucidated. Sedentary behavior may operate through various metabolic and inflammatory pathways [33, 34]. For example, epidemiologic studies using objective measures to assess sedentary time have reported significant positive associations between total time spent sedentary and blood glucose and lipid levels [35, 36]. Findings from an experimental study revealed reduced peripheral insulin sensitivity in participants who lowered their daily steps from 10,501 to 1,344 steps per day during a 2-week period [37]. Furthermore, observational studies [38–40] and randomized clinical trials [41, 42] show that reducing TV time and time spent sedentary decreases the risk of obesity and type 2 diabetes. Animal studies demonstrate that physical inactivity suppresses lipoprotein lipase (LPL) activity, which is involved in the uptake of free fatty acids and triglycerides into skeletal muscle and production of high-density lipoprotein (HDL) cholesterol [43–45]. Glucose uptake may also be lowered by reduced muscle contraction through blunted translocation of GLUT-4 glucose transporters to the skeletal muscle cell surface [46]. Increased levels of glucose, triglycerides, and free fatty acids can produce excess free radicals and promote endothelial dysfunction, inflammation, hypercoagulability, and other atherogenic changes, which may predispose to coronary heart disease [46–49]. Moreover, time spent sedentary, particularly while watching TV is associated with greater food consumption and energy intake [50], lower energy expenditure [51], and weight gain [52]. These variables are associated with increased risk of mortality [53–55].

For physical activity, the potential mechanisms underlying the risk of mortality are suspected to involve improvements in chronic inflammation, immune function, and antioxidant enzyme systems, changes in sex hormones, and weight reduction [56–58]. Increased exercise training may alter cholesterol metabolism, particularly HDL-cholesterol metabolism, with low levels of HDL-cholesterol responding positively to exercise training [59]. Physical activity has been shown to reduce thrombotic variables and to decrease overall cardiovascular disease incidence and mortality [60–62]. Moreover, exercise may reduce levels of estrogen and androgens [63–65], which are positively related to cancer incidence [63, 66, 67] and may partly contribute to risk of cancer mortality [68, 69].

### Strengths and limitations

A particularly favorable feature of the present study is the objective assessment of sedentary behavior and physical activity, which is considered more accurate than self-report methods [17–21]. Further strengths of our study include the nationally representative sample of middle-aged to older adults, its prospective design, and the ability to carefully address the impact of potential confounding.

Several limitations of our study merit comment, including the short duration of follow-up, which may have led to reverse causation. Although we conducted extensive sensitivity analyses by adjusting for health conditions or disability and restricting the data to a relatively healthy population, it remains likely that we overestimated the effects of sedentary time and moderate to vigorous physical activity on mortality risk in our study because pre-existing but undiagnosed chronic disease may have influenced levels of sedentary time and physical activity at baseline. Because the physical activity data were skewed, the median value for moderate to vigorous physical activity of 6.60 min/day was not very high in the dataset. Although we carefully adjusted for a wide range of potential confounding variables, unmeasured or unknown confounding may have partly accounted for the reported associations.

A further shortcoming of this study is that accelerometers do not allow differentiating between postures of low energy expenditure (e.g., sitting versus standing). Moreover, using accelerometer-based data, we were unable to distinguish between different types of sedentary time (i.e., watching TV, working, driving, or socializing) and different types of physical activity (i.e., recreational, household, transportation, or occupational physical activity), which may differentially affect risk for mortality. In addition, accelerometers are not sensitive to detecting upper body movements, biking, or swimming, which could have led us to underestimate physical activity levels of study participants.

## Conclusion

In conclusion, in this population of middle-aged to older individuals, we found that both high levels of sedentary time and low levels of moderate to vigorous physical activity were independent predictors of early mortality from any cause. Whether greater moderate to vigorous physical activity compensates for the increased mortality risk associated with a high volume of sedentary time requires further investigation in larger studies with longer follow-up. While current recommendations and intervention approaches focus on adequate levels of physical activity for protection of health, individual-level and public health efforts to reduce the time spent sedentary have been given less weight [70, 71] and only a few countries have incorporated sedentary behavior recommendations in their guidelines [72–74]. Physical activity guidelines may need to be expanded by incorporating recommendations to reduce time spent in sedentary behaviors.
